# Cardiovascular disease risk in people of African ancestry with HIV in the United Kingdom

**DOI:** 10.1111/hiv.13706

**Published:** 2024-08-29

**Authors:** Stephanie Ko, Lourdes Dominguez‐Dominguez, Zoe Ottaway, Lucy Campbell, Julie Fox, Fiona Burns, Lisa Hamzah, Andrew Ustianowski, Amanda Clarke, Stephen Kegg, Sarah Schoeman, Rachael Jones, Sarah L. Pett, Jonathan Hudson, Frank A. Post

**Affiliations:** ^1^ King's College Hospital NHS Foundation Trust London UK; ^2^ Berkshire Healthcare NHS Foundation Trust Slough UK; ^3^ King's College London London UK; ^4^ Guys and St Thomas's NHS Foundation Trust London UK; ^5^ Royal Free London NHS Foundation Trust London UK; ^6^ Institute for Global Health University College London London UK; ^7^ St Georges University Hospital NHS Foundation Trust London UK; ^8^ Manchester University NHS Foundation Trust Manchester UK; ^9^ University Hospitals Sussex NHS Foundation Trust Brighton UK; ^10^ Lewisham and Greenwich NHS Trust London UK; ^11^ Leeds Teaching Hospitals NHS Trust Leeds UK; ^12^ Chelsea and Westminster NHS Foundation Trust London UK; ^13^ Central and North West London NHS Foundation Trust London UK

**Keywords:** Black, BMI, CVD risk, GLP‐1 agonists, HIV, obesity, statins

## Abstract

**Objectives:**

Our objective was to describe the prevalence of cardiovascular disease (CVD) risk factors in people of African ancestry with HIV in the UK.

**Methods:**

We conducted a cross‐sectional analysis of CVD risk factors in Black people with HIV aged ≥40 years and estimated the 10‐year CVD risk using QRISK®3‐2018. Correlations between body mass index (BMI) and CVD risk factors were described using Pearson correlation coefficients, and factors associated with 10‐year CVD risk ≥5% were described using logistic regression.

**Results:**

We included 833 Black people with HIV and a median age of 54 years; 54% were female, 50% were living with obesity (BMI ≥30 kg/m^2^), 61% had hypertension, and 19% had diabetes mellitus. CVD risk >5% ranged from 2% in female participants aged 40–49 years to 99% in men aged ≥60 years, and use of statins ranged from 7% in those with CVD risk <2.5% to 64% in those with CVD risk ≥20%. BMI was correlated (*R* ^2^ 0.1–0.2) with triglycerides and diastolic blood pressure in women and with glycated haemoglobin, systolic and diastolic blood pressure, and total:high‐density lipoprotein (HDL) cholesterol ratio in men. In both female and male participants, older age, blood pressure, diabetes mellitus, and kidney disease were strongly associated with CVD risk ≥5%, whereas obesity, total:HDL cholesterol, triglycerides, and smoking status were variably associated with CVD risk ≥5%.

**Conclusions:**

We report a high burden of CVD risk factors, including obesity, hypertension, and diabetes mellitus, in people of African ancestry with HIV in the UK. BMI‐focused interventions in these populations may improve CVD risk while also addressing other important health issues.

## INTRODUCTION

Cardiovascular disease (CVD) is a major concern to people of African ancestry with HIV. In these populations, CVD risk factors, including hypertension, diabetes mellitus, and chronic kidney disease (CKD), are highly prevalent [1], and major CVD events such as heart failure [2] and stroke [3] are notably more common in African Americans than in white or Hispanic populations. People with HIV are at an increased risk of CVD, including heart failure [4], myocardial infarction, and stroke [5]. This increased atherosclerotic CVD risk among people with HIV may relate to traditional CVD risk factors such as smoking and dyslipidaemia as well as immune activation and vascular inflammation [6]. The burden of HIV‐associated CVD is greatest in the countries of sub‐Saharan Africa most severely affected by the HIV pandemic [5].

REPRIEVE, a large, randomized controlled clinical trial, showed that pitavastatin reduced the risk of major CVD events by 35% in people with HIV at low‐to‐moderate CVD risk [7]. Consequently, the British HIV Association recommends that all people living with HIV aged ≥40 years should be offered a statin for primary prevention of CVD, irrespective of lipid profile or estimated CVD risk [8]. Although REPRIEVE had global representation, the incidence of CVD endpoints in sub‐Saharan African populations was very low (<2/1000 person‐years of follow‐up), suggesting that around 300 African people with HIV at low‐to‐moderate CVD risk would need to be treated for 5 years to prevent one major CVD event.

An additional target for CVD risk reduction in people of African ancestry is body mass index (BMI) [9]. Obesity is an established CVD risk factor [10, 11], which is only partially explained by its effects on blood pressure, glycaemia, and lipids [12]. Effective treatments for obesity, such as glucagon‐like peptide 1 (GLP‐1) analogues have become available and have been associated with a 14%–20% reduction in major CVD events [13, 14, 15], although this has not been established in people with HIV.

In light of the REPRIEVE trial [7] and its impact on clinical guidance, it is important to understand CVD risk in people with HIV from sub‐Saharan Africa and the Caribbean to deliver effective primary CVD prevention. We sought to describe overall CVD risk, use of statins, and the prevalence and control of various CVD risk factors in people of African ancestry with HIV in the UK. We explored the relationship between BMI and other CVD risk factors to assess possible benefits of a BMI‐lowering strategy in this population.

## METHODS

We used data from the GEN‐AFRICA (Genetic Determinants of Kidney Disease in people of African ancestry with HIV; NCT05685810) study [1], and two GEN‐AFRICA sub‐studies (CKD‐AFRICA and COVID‐AFRICA) [16, 17]. The GEN‐AFRICA study was open to all individuals of Black ethnicity with HIV at 15 sites across the UK; the COVID‐AFRICA study was open to all GEN‐AFRICA participants who remained in HIV care at 11 sites during the COVID‐19 pandemic, and the CKD‐AFRICA study was open to GEN‐AFRICA participants aged 30–65 years who received HIV care at three South London sites. All three studies received approval from a National Health Service Research Ethics Committee (18/LO/0234, 20/LO/0946 and 21/ES/0047) and the Health Research Authority (IRAS 239895, 278 244 and 294 887), and all participants provided written informed consent.

The current analyses are restricted to participants aged ≥40 years (i.e., the age group of the REPRIEVE trial population) who had three standardized blood pressure, height, and weight measurements recorded as part of their CKD‐AFRICA or COVID‐AFRICA study visits conducted between September 2020 and November 2022 (i.e., before publication of the REPRIEVE trial findings). At these visits, clinical information, including diagnoses of hypertension, diabetes mellitus, and atherosclerotic cardiovascular disease (ischaemic heart disease, stroke, and peripheral vascular disease) was obtained through questionnaires and medical records review. A detailed drug history, including medications to treat hypertension, diabetes, CVD, and dyslipidaemia, was obtained. Non‐fasting blood tests, including HIV viral load, kidney function, glycated haemoglobin (HbA1c), total‐cholesterol, high‐density lipoprotein (HDL) cholesterol, and triglycerides, were performed unless recent measures were available. Smoking status was previously ascertained during the GEN‐AFRICA study visit (2018–2020).

CVD risk was estimated using QRISK®3‐2018 (https://www.qrisk.org/), a CVD risk prediction tool based on UK primary care records and validated for use in ethnically diverse populations [18], with data for age; gender; ethnicity; smoking status; diagnoses of diabetes, CKD, atrial fibrillation, rheumatoid arthritis, systemic lupus erythematosus, migraines, severe mental illness, or erectile dysfunction; use of anti‐hypertensive, antipsychotic, or corticosteroid medication; total:HDL cholesterol ratio; systolic blood pressure; height; and weight. As we lacked information on CVD family history, smoking intensity, and type of diabetes, all current smokers were considered moderate smokers (10–19 cigarettes per day), and all participants with diabetes were considered to have type 2 diabetes. CKD was defined by an estimated glomerular filtration rate <60 mL/min/1.73 m^2^.

CVD risk was described for the overall population and stratified by age and gender. Current use of statins was described by CVD risk stratum. Correlations between BMI and HbA1c, triglycerides, systolic and diastolic blood pressure, and total:HDL cholesterol ratio were described using Pearson correlation coefficients. We evaluated the degree of control of these CVD risk factors by calculating the proportions of participants with optimal BMI (<25 kg/m^2^), without pre‐diabetes or diabetes (HbA1c <42 mmol/mol), normal systolic and diastolic blood pressure (<140 and <90 mm Hg), optimal total:HDL cholesterol ratio (<3.5), and triglycerides (<1 mmol/L); we considered measures above these thresholds to be raised or sub‐optimal and considered BMI ≥30 kg/m^2^, HbA1c ≥48 mmol/mol, systolic and diastolic blood pressure ≥160 and ≥100 mm Hg, total:HDL‐ cholesterol ratio ≥6, and triglycerides ≥2.3 mmol/L to be high or unfavourable.

Demographic and clinical characteristics of the study population, stratified by gender, were described and compared using the chi‐squared test (or Fisher's exact test for variables with frequency <5 in either subgroup) for categorical data, or the Kruskal–Wallis test for continuous data. Logistic regression was used to examine the associations between individual CVD risk factors and a 10‐year CVD risk ≥5%. The observed association between BMI ≥30 kg/m^2^ and CVD risk ≥5% in univariable association in women was adjusted in multivariable analysis for demographic parameters with *p* < 0.1. All statistical analyses were performed using R (R Foundation, Vienna, Austria; version 4.2.1).

## RESULTS

**FIGURE 1:**
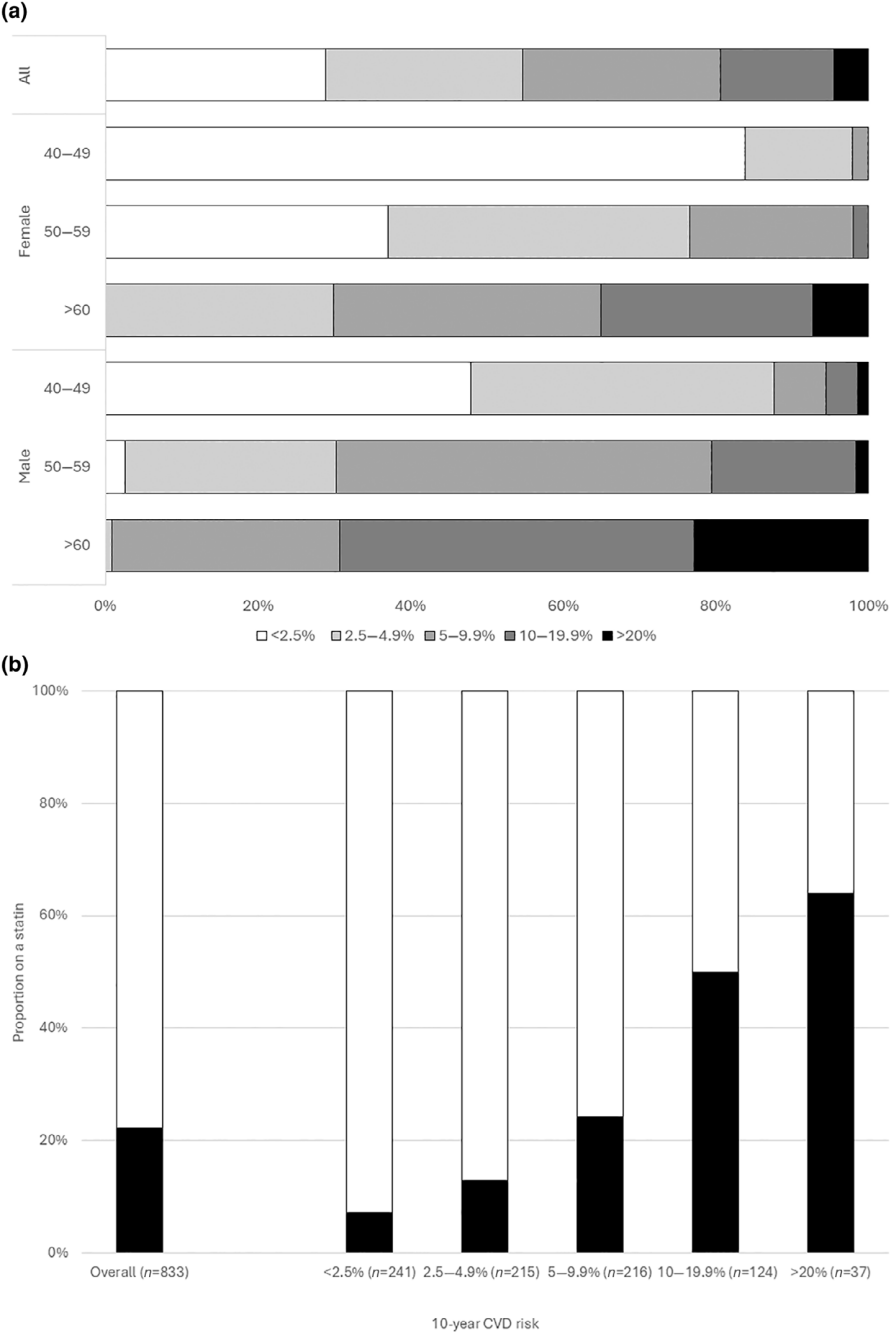
(a) Distribution of 10‐year cardiovascular disease risk in study participants, overall and stratified by sex and age and (b) the proportion in each stratum who reported taking lipid‐lowering therapy. Black bars indicate statins.

We included 833 participants with a median age of 54 years (interquartile range [IQR] 49–60); 54% were female, and 86% were born in sub‐Saharan Africa or the Caribbean. Most had longstanding (median 17 years [IQR 12–21]) and well‐controlled HIV (viral load <200 copies/mL; 89%). Male participants were slightly older and more often current smokers, had higher total:HDL cholesterol and triglyceride levels and systolic and diastolic blood pressures, and more frequently had CKD and diabetes mellitus and higher CVD risk scores, whereas female participants had higher BMIs (Table 1). Antiretroviral medications are described in Table S1. Overall, 45% of participants had a CVD risk >5%, ranging from 2% in female participants aged 40–49 years to 99% in men aged ≥60 years, and 19% were at moderate (10%–20%) or high (≥20%) CVD risk (Figure 1a). A total of 178 participants (22%) reported taking a statin; the proportion taking a statin increased with greater CVD risk, ranging from 7% to 64% in those with CVD risk <2.5% and ≥20%, respectively (Figure 1b).

**TABLE 1:** Participant characteristics.

Characteristic	Overall (*N* = 833)	Female (*N* = 451)	Male (*N* = 382)	*p* value
Demographic and HIV parameters				
Age, years	54 (49–60)	53 (48–59)	56 (51–61)	**<0.001**
Region of birth				**<0.001**
Sub‐Saharan Africa	656 (79.6)	385 (86.1)	271 (71.9)	
Caribbean	56 (6.8)	15 (3.4)	41 (10.9)	
UK/other	112 (13.6)	47 (10.5)	65 (17.2)	
Years since HIV infection diagnosis	17 (12–21)	18 (13–21)	16 (11–20)	**<0.001**
CD4 cell count (cells/mm^3^)	558 (404–748)	604 (441–787)	515 (375–675)	**<0.001**
HIV RNA <200 copies/mL	743 (89.1)	408 (90.4)	335 (87.6)	0.25
HBV (HBV surface antigen)	24 (2.9)	14 (3.1)	10 (2.6)	0.833
HCV (anti‐HCV)	3 (0.4)	1 (0.2)	2 (0.5)	0.885
CVD risk factors				
BMI (kg/m^2^)	30 (26–34)	32 (28–36)	28 (25–31)	**<0.001**
Current smoker	69 (8.3)	15 (3.3)	54 (14.1)	**<0.001**
Total:HDL cholesterol ratio	3.3 (2.7–4.0)	3.1 (2.6–3.8)	3.5 (2.9–4.3)	**<0.001**
Triglycerides (mmol/L)	1.1 (0.8–1.6)	1.0 (0.8–1.4)	1.3 (0.9–1.8)	**<0.001**
Hypertension	504 (60.5)	250 (55.4)	254 (66.5)	**0.001**
Mean systolic BP (mmHg)	133 (120–145)	129 (115–142)	137 (126–148)	**<0.001**
Mean diastolic BP (mmHg)	83 (76–91)	81 (75–91)	84 (77–92)	**0.007**
HbA1c (mmol/mol)	39 (36–43)	39 (36–43)	40 (36–44)	0.36
eGFR <60 mL/min/1.73 m^2^	103 (12.4)	39 (8.6)	64 (16.8)	**<0.001**
History of IHD, stroke or PVD	24 (2.9)	13 (2.9)	11 (2.9)	1.00
Diabetes mellitus	161 (19.3)	73 (16.2)	88 (23.0)	**0.016**
10‐year CVD (Q) risk	4.3 (2.1–8.3)	2.7 (1.4–5.1)	7.2 (4.1–11.1)	**<0.001**
On a statin	178 (22.2)	77 (17.9)	101 (27.2)	**0.002**

*Note*: Bold indicates statistically significant values.

Abbreviations: BMI, body mass index; BP, blood pressure; CVD, cardiovascular disease; eGFR, estimated glomerular filtration rate; HbA1c, glycated haemoglobin; HBV, hepatitis B virus; HCV, hepatitis C virus; HDL, high‐density lipoprotein; IHD, ischaemic heart disease; PVD, peripheral vascular disease.

Data are presented as *N* (%) or median (interquartile range) unless otherwise indicated.

Obesity, hypertension, and diabetes mellitus were highly prevalent, affecting 62%, 55%, and 16% of female and 36%, 67%, and 23% of male participants, respectively. By contrast, smoking was relatively uncommon, with 8% being current smokers. We examined correlations between BMI and other CVD risk factors and found a greater BMI to be associated with higher HbA1c, triglycerides, systolic and diastolic blood pressure, and total:HDL cholesterol ratio (Figure S1). In female participants, we observed significant correlations between BMI and triglycerides and between BMI and diastolic blood pressure; in male participants, BMI was significantly correlated with HbA1c, systolic and diastolic blood pressure, and total:HDL cholesterol ratio, albeit at relatively low *R* ^2^ values ranging from 0.1 to 0.2 (Table 2).

**TABLE 2:** Correlation between body mass index and other cardiovascular risk factors.

Risk factor	Female		Male	
	*R*‐value	*p*‐value	*R*‐value	*p*‐value
HbA1c	0.07	0.12	**0.15**	**<0.005**
Triglycerides	**0.13**	**<0.01**	0.06	0.24
Systolic BP	0.07	0.16	**0.11**	**0.04**
Diastolic BP	**0.10**	**0.03**	**0.13**	**0.01**
TC:HDL cholesterol	0.08	0.10	**0.18**	**<0.001**

*Note*: Pearson correlation coefficients and corresponding *p* values are displayed. Bold indicates statistically significant values.

Abbreviations: BP, blood pressure; HbA1c, glycated haemoglobin; HDL, high‐density lipoprotein; TC, total cholesterol.

**FIGURE 2:**
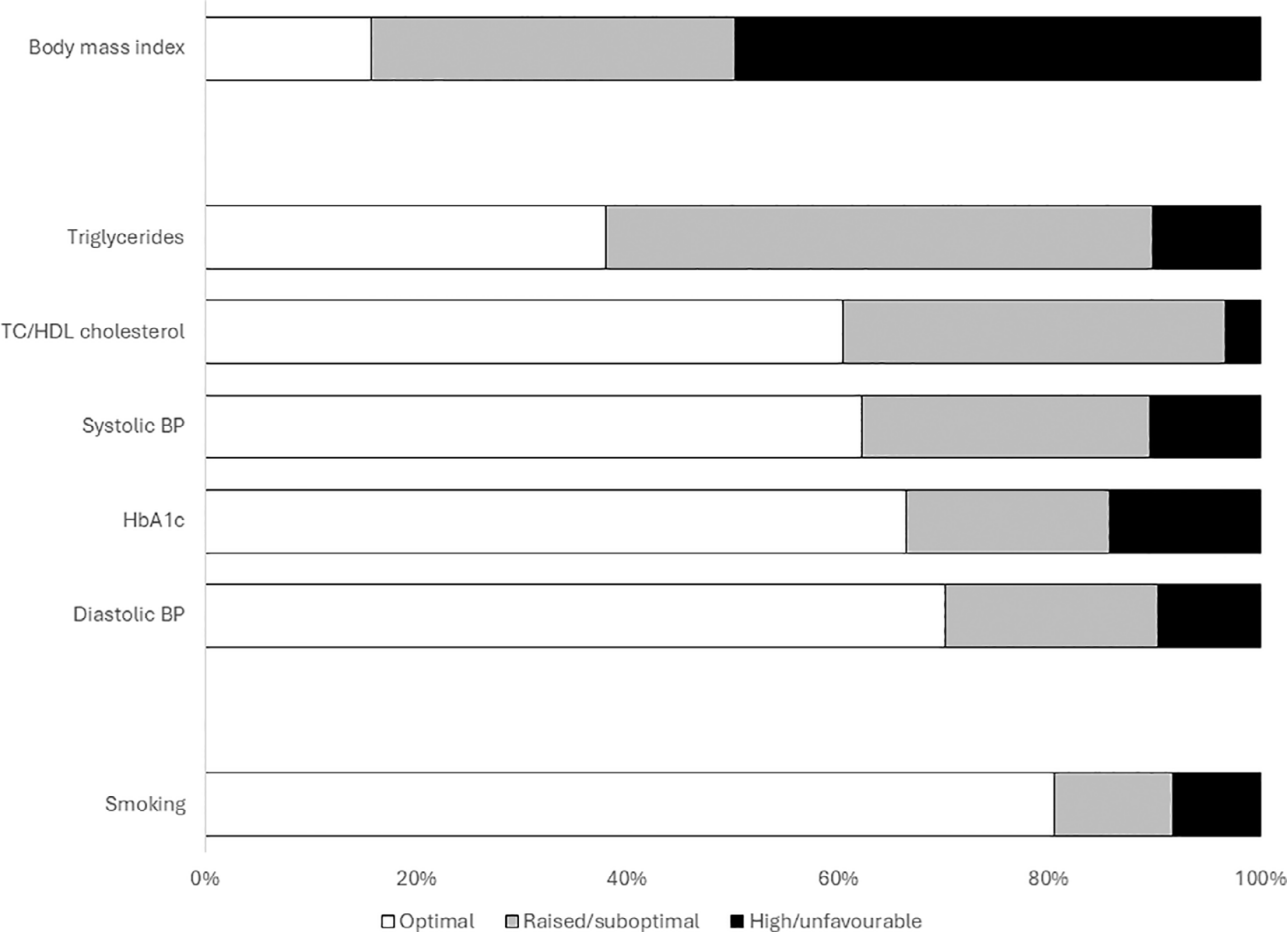
Opportunities for optimization of cardiovascular disease risk factors. Proportion of study participants with optimal, raised/suboptimal, and high/unfavourable status, respectively defined as body mass index <25, 25.0–29.9, and ≥30 kg/m^2^; non‐fasting triglycerides <1, 1–2.2, and ≥2.3 mmol/L; total cholesterol (TC):high‐density lipoprotein (HDL) cholesterol ratio <3.5, 3.5–5.9, and ≥6; systolic blood pressure (BP) <140, 140–159, and ≥160 mm Hg; diastolic BP <90, 90–99, and ≥100 mm Hg; glycated haemoglobin (HbA1c) <42, 42–47, and ≥48 mmol/mol; and smoking status (never, ex‐smoker, and current smoker).

We stratified participants according to whether they had optimal, raised/suboptimal, or high/unfavourable status for various CVD risk factors (Figure 2). A very high proportion of participants had suboptimal BMI (≥25 kg/m^2^ [84%]), lipid profiles (triglycerides ≥1 mmol/L [62%], total:HDL cholesterol ratio ≥3.5 [39%]), blood pressure (systolic blood pressure ≥140 mm Hg [38%], diastolic blood pressure ≥90 mm Hg [30%]), and HbA1c (≥42 mmol/mol [34%]). Even at stringently defined cut‐offs, 10% of participants had high systolic and/or diastolic blood pressures and/or triglyceride levels, 3% had high total:HDL ratios, and 14% had high HbA1c measurements.

In both female and male participants, older age, diabetes, and CKD were strongly associated with a CVD risk ≥5% (Table S2). Additionally, in female participants, region of birth (UK/other vs. Africa or the Caribbean), BMI ≥30 kg/m^2^, systolic and diastolic blood pressure, total:HDL cholesterol, triglycerides, and smoking status were strongly associated with CVD risk >5%. The association between BMI >30 kg/m^2^ and CVD risk ≥5% (odds ratio 1.74 [95% confidence interval 1.07–2.92]) was minimally affected but no longer statistically significant after adjustment for demographic parameters (adjusted odds ratio 1.70 [95% confidence interval 0.92–3.21]). Among male participants, only systolic and diastolic blood pressure were associated with CVD risk ≥5%.

## DISCUSSION

We report on the CVD risk profiles among people of African ancestry with HIV in the UK. CVD risk was low in both women and men aged <50 years but increased substantially thereafter. The use of statins was low across the CVD risk spectrum, even among those at moderate or high CVD risk. Obesity and obesity‐related CVD risk factors such as type 2 diabetes, CKD, hypertension, and hypertriglyceridaemia were highly prevalent in this cohort, highlighting opportunities for a BMI‐focused approach to CVD risk reduction in this population.

The benefits of statins are most pronounced in those at moderate or high (>10%) CVD risk, in whom a 35% relative reduction translates to a substantial absolute risk reduction. When the British HIV Association recommendation to offer statins to all people living with HIV aged ≥40 years, irrespective of CVD risk [8], is applied to our study population, large numbers of women and men at low or very low CVD risk would be encouraged to initiate statins with little effect on short‐ and medium‐term health outcomes. Consistent with data from the general population [19], a large proportion of those at moderate to high CVD risk (for whom statins were recommended at the time of the study visits) were not on statins, and a focus on this subset remains prudent. Reasons for non‐use of statins, which may include lack of access to general practitioner services or concerns about an increase in pill burden (especially if already taking antihypertensive or hypoglycaemic medications), side effects, perceived limited benefit, or – where these medications are not provided free of charge – recurring cost. Some of these factors may be even greater barriers to the widespread uptake of statins in populations at lower CVD risk, in whom the balance between necessity and concerns may be less in favour of uptake. Further research should provide insight into CVD risk thresholds above which medications to reduce CVD risk are acceptable to participants, the number of medications participants would be willing to take to reduce their CVD risk, and whether specific risk factors such as systolic hypertension are perceived as a greater priority.

The lower CVD event rate in the statin arm of the REPRIEVE study was largely driven by participants from high‐income countries [7]. The GEN‐AFRICA participants differ in several important ways from the REPRIEVE trial population, with lower smoking rates (8% vs. 25%), a higher median BMI (30 vs. 26 kg/m^2^), and higher rates of hypertension (60% vs. 36%). Although the REPRIEVE trial was not designed to evaluate the effects of statins in sub‐groups, it is concerning that no benefit was observed in people with hypertension or those from the Caribbean/Latin America. Although it is implausible that the benefits of statins would not extend to these populations, the possibility of a somewhat smaller benefit in such individuals, and the low absolute risk of CVD events in the African participants, might need to be considered and discussed in conjunction with the observed 35% increase in new‐onset diabetes mellitus in the pitavastatin arm of the trial when people of African and Caribbean ethnicity with HIV, and those with hypertension, are being offered statins to reduce their CVD risk.

The high rates of obesity in our study participants, the disadvantageous relationship between obesity and several CVD risk factors, and the additional CVD risk conferred by obesity suggest that a BMI‐focused CVD risk reduction strategy may be effective and target several risk factors concurrently. Semaglutide and tirzepatide are examples of GLP‐1 agonist treatments for obesity; clinical trials in which these medications were administered together with lifestyle interventions for 68–72 weeks, and from which people with HIV were excluded, have shown substantial reductions in body weight (15%–21%) accompanied by reductions in HbA1c (12.6%), systolic blood pressure (6.2–7.2 mm Hg), diastolic blood pressure (2.8–4.8 mm Hg), triglycerides (0.31–0.64 mmol/L), and non‐HDL cholesterol (0.21–0.25 mmol/L) and by increases in HDL cholesterol (0.06–0.21 mmol/L) [20, 21]. In high‐risk populations such as people with type 2 diabetes or pre‐existing CVD and a raised BMI, a median exposure of 44 weeks (tirzepatide) to 40 months (semaglutide) was associated with a 20% reduction in major CVD events [14, 15]. GLP‐1 agonist‐induced weight reduction may provide additional health benefits, including a reduced risk of kidney disease progression in people with diabetes [22, 23], an improvement of obstructive sleep apnoea symptoms [24], and – by improving physical function [20, 21] – potentially a reduced risk of venous thrombosis. Of note, the proportion of participants of Black ethnicities in these studies was low (3.5%–7.9%), and further studies in these populations are warranted.

In terms of non‐pharmacological interventions, the low rates of current smoking in our study population provide relatively few opportunities to improve CVD risk through smoking‐cessation strategies. Public health messaging might highlight this remarkable achievement while discouraging younger people of African and Caribbean ethnicities from taking up smoking. As both abacavir and darunavir have been associated with an increased risk of myocardial infarction in observational cohort studies [25, 26], CVD risk may be reduced by switching to alternative antiretroviral medications.

The strengths of this study include a large sample size of both women and men and comprehensive data on most CVD risk factors, including BMI, HbA1c, and standardized blood pressure measurements, and use of a risk‐prediction tool established and validated for Black‐African and Black Caribbean populations living in the UK. We acknowledge some limitations, including the use of recent rather than current smoking status, a lack of information on smoking intensity and family history of CVD, and use of BMI rather than waist circumference [27] or specific measures of visceral adiposity. Although Q‐risk is validated to assess CVD risk for primary prevention purposes, our cohort includes a small number (*n* = 24; 2.9%) of participants with a history of atherosclerotic CVD, and Q‐risk may underestimate CVD risk in people with HIV [18].

In summary, we report a high burden of CVD risk factors, including obesity, in people of African ancestry with HIV in the UK. Our data suggest that a BMI‐focused approach may improve CVD risk while addressing other important health issues in these populations, such as diabetes and CKD. Preliminary observations suggest a high level of awareness among GEN‐AFRICA participants of the adverse health effects of obesity, a desire to have a lower BMI, and a strong interest in GLP‐1 agonists to help achieve this (FAP, unpublished observations). Although statins have an important and established role in reducing CVD risk in high‐income countries, further studies to define preferred and optimal CVD risk reduction strategies in Black populations with HIV, especially those living in low‐and middle‐income countries, are warranted.

## AUTHOR CONTRIBUTIONS

The study was designed by FAP. SKo, LD‐D, ZO, LC, and FAP analyzed the data. JF, FB, LH, AU, AC, SKe, SS, RJ and SLP were site principal investigators, coordinating recruitment and data collection at their sites. JH and FAP interpreted the findings. FAP wrote the first draft of the manuscript with input from JH and site principal investigators. All authors revised and approved the final version of the manuscript.

## CONFLICT OF INTEREST STATEMENT

JF has received grants from GlaxoSmithKline. FB has received grants and personal fees from Gilead Sciences. AU has received personal fees from Generate, Gilead Sciences, ViiV Healthcare/GlaxoSmithKline, Merck/MSD, Moderna, and Pfizer. AC has received grants and personal fees from Gilead Sciences, grants and personal fees from ViiV Healthcare/GlaxoSmithKline, and grants and personal fees from MSD. SS has received personal fees from ViiV Healthcare and grants from Gilead Sciences, ViiV Healthcare/GlaxoSmithKline, and MSD. RJ has received grants from Gilead Sciences and personal fees from ViiV Healthcare. SLP has received grants from Janssen‐Cilag, ViiV Healthcare, and Gilead Sciences. FAP has received personal fees and grants from Gilead Sciences, ViiV Healthcare/GlaxoSmithKline, and MSD. SKo, LD‐D, ZO, LC, LH, SKe, and JH have no competing interests.

## INSTITUTIONAL REVIEW BOARD STATEMENT

The study was approved by the National Health Service Research Ethics Committee (18/LO/0234, 20/LO/0946 and 21/ES/0047) and the Health Research Authority (IRAS 239895, 278 244 and 294 887).

## Supporting information


**Figure S1.** Correlation between body mass index (BMI) and other cardiovascular disease (CVD) risk factors.
**Table S1.** Antiretroviral therapies.
**Table S2.** Factors associated with 10 year cardiovascular disease (CVD) risk ≥5% among female and male participants aged 40–65 years.

## Data Availability

The database contains personal and sensitive information and is therefore not publicly available. Access to the study data and/or samples is governed by the National Health Service data access policy and those of King's College Hospital NHS Foundation Trust, the study sponsor. The GEN‐AFRICA, CKD‐AFRICA, and COVID‐AFRICA studies are open to collaborations, and all requests from researchers who meet the criteria for access to fully anonymized patient‐level data will be considered. Concepts can be submitted for review to the principal investigator (Prof. Frank Post; email: frank.post@kcl.ac.uk).

## APPENDIX A GEN‐AFRICA STUDY GROUP

University Hospitals Sussex NHS Foundation Trust, Brighton.

Amanda Clarke (PI), Marion Campbell, Alyson Knott, Lisa Barbour, Vittorio Trevitt.

**Chelsea and Westminster NHS Foundation Trust, London:**

Rachael Jones (PI), Alex Schoolmeesters, Shoreh Ghazali.

**Guys and St Thomas's NHS Foundation Trust, London:**

Julie Fox (PI), Anele Waters, Mariusz Racz, Louise Terry, Kathy Arbis.

**King's College Hospital NHS Foundation Trust, London:**

Frank Post (CI and PI), Zoe Ottaway, Beatriz Santana‐Suarez, Leigh McQueen, Lucy Campbell, Bee Barbini, Emily Wandolo, Rachel Hung, Luella Hanbury, Sarah Barber, Chris Taylor, Mary Poulton, Holly Middleditch, Itty Samuel, Candice McDonald, Vladimir Kolodin, Laura Cechin, Larissa Mulka, Amy John, Sally Hawkins, Madelaine Angus, Tim Appleby, Katherine Bainbridge, Hayley Cheetham, Julie Barker, Kate Childs, Rousell Roberts, Nivenjit Kaur, Lisa Shorrock, Kate El Bouzidi, Nisha Patel, Amelia Oliveira, Ayoma Ratnappuli, Kate Flanagan, Daniel Trotman, Nisha Mody, Michael Brady, Killian Quinn, Elizabeth Hamlyn, Gurjinder Sandhu, Verity Sullivan, Naomi Fitzgerald.

**Leeds Teaching Hospitals NHS Trust, Leeds:**

Sarah Schoeman (PI), Gary Lamont, Natasha Calder‐Smith, Tadas Mazeika, Kiran Chana.

**North Manchester General Hospital, Manchester:**

Andrew Ustianowski (PI), Gabriella Lindergard, Jan Flaherty, Valerie George, Denise Kadiu, Alice Hendy, Kevin Kuriakose.

**Mortimer Market Centre; Central and North West London NHS Foundation Trust, London:**

Sarah Pett (PI), Mah Jabeen Qamar, Jose Paredes Sosa, Konstantina Tetorou, Michelle Beynon, Adesola Yinka‐Ogunleye, Gosala Gopalakrishnan, Irfaan Maan, Claire Mullender.

**Queen Elizabeth Hospital, Woolwich and Lewisham and Greenwich NHS Trust, London:**

Stephen Kegg (PI), Chloe Saad, Rosa Harrington, Kirsty Cunningham, Mandy Lewis, Melanie Rosenvinge, Claudia Adade, Shelley Campbell.

**Royal Free London NHS Foundation Trust, London:**

Fiona Burns (PI), Jonathan Edwards, Tom Fernandez, Qayo Egeh, Megan Bailey, Katie Spears.

**St George's University Hospital NHS Foundation Trust, London:**

Lisa Hamzah (PI), Catherine Cosgrove, Hannah Laurence, Ameen Rahman, Katie Toler.

**Africa Advocacy Foundation:**

Denis Onyango.
