# Supplementary material for: Cardiovascular disease risk in people of African ancestry with HIV in the United Kingdom
Source: HIV Med. 2024 Aug 29;25(12):1289–97. doi: 10.1111/hiv.13706 (PMC11608579; doi:10.1111/hiv.13706)
Supplement: Supplementary file 1 — Figure S1. Correlation between body mass index (BMI) and other cardiovascular disease (CVD) risk factors. Table S1. Antiretroviral therapies. Table S2. Factors associated with 10 year cardiovascular disease (CVD) risk ≥5% among female and male participants aged 40–65 years. [file HIV-25-1289-s001.docx]

**Figure S1: Correlation between BMI and other CVD risk factors**


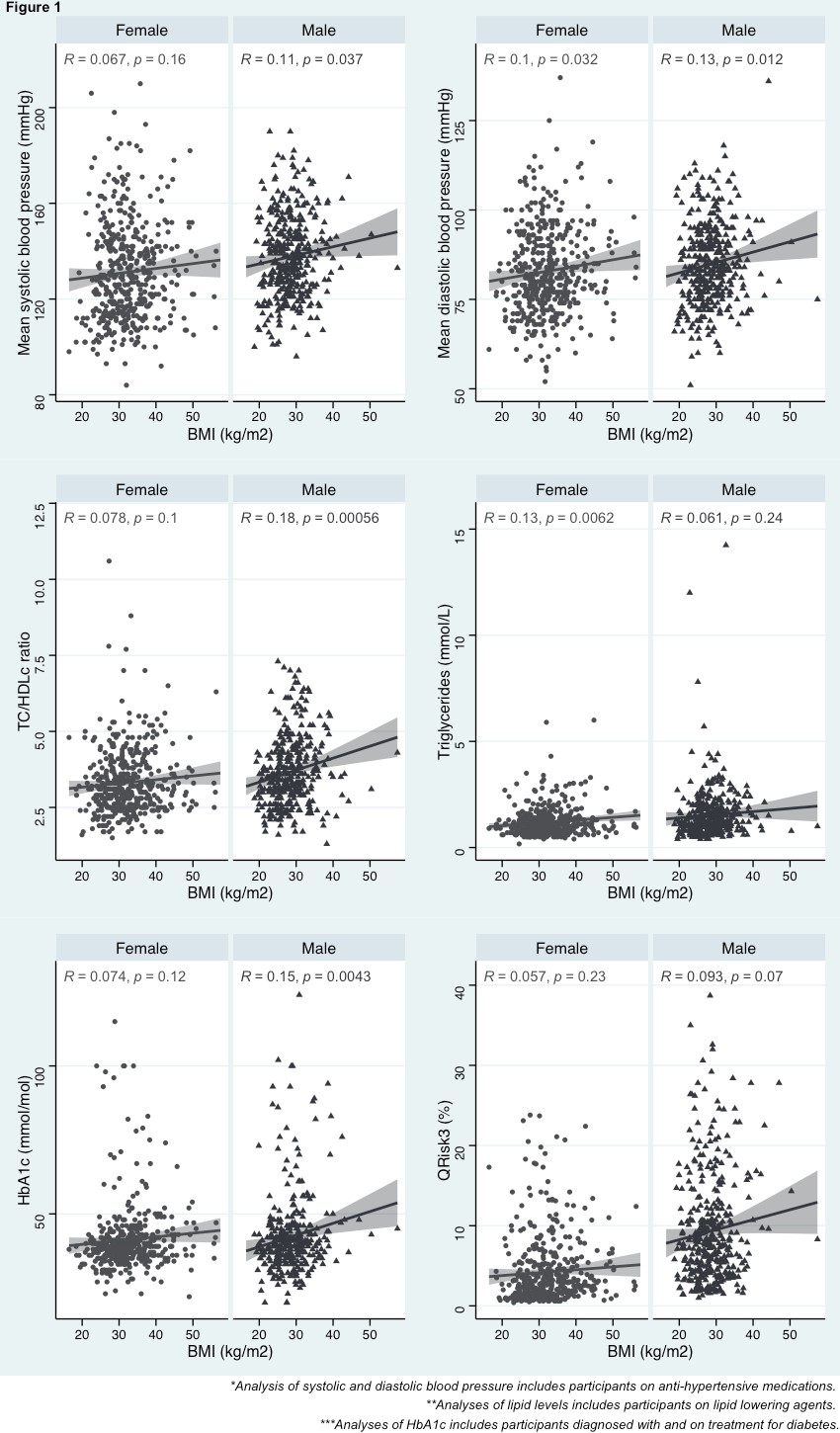


**Table S1 Antiretroviral therapies**

|  |  | **Overall (N=833)** | **Female (N=451)** | **Male (N=382)** | **p-value** |
| --- | --- | --- | --- | --- | --- |
|  |  |  |  |  |  |
| **ART backbone** |  |  |  |  | 0.012 |
| No TDF, TAF or Abacavir | N (%) | 93 (11.4) | 48 (11.0) | 45 (11.9) |  |
| Tenofovir-DF | N (%) | 279 (34.1) | 143 (32.6) | 136 (35.9) |  |
| Tenofovir alafenamide | N (%) | 213 (26.1) | 102 (23.3) | 111 (29.3) |  |
| Abacavir | N (%) | 232 (28.4) | 145 (33.1) | 87 (23.0) |  |
|  |  |  |  |  |  |
| **ART third drug** |  |  |  |  | 0.86 |
| NNRTI | N (%) | 298 (36.0) | 164 (36.9) | 134 (35.1) |  |
| INSTI | N (%) | 340 (41.1) | 180 (40.4) | 160 (41.9) |  |
| Protease inhibitor | N (%) | 189 (22.9) | 101 (22.7) | 88 (23.0) |  |

ART=Antiretroviral therapy; TDF=tenofovir disoproxil, TAF=tenofovir alafenamide

NNRTI=Non-nucleoside reverse-transcriptase inhibitor;

INSTI=integrase strand-transfer inhibitor

**Table S2: Factors associated with 10 year CVD risk >5% among female and male participants aged 40-65 years**

|  | **Female 40-65 years (N=426)** | | | | | | |  | | **Male 40-65 years (N=342)** | | | |
| --- | --- | --- | --- | --- | --- | --- | --- | --- | --- | --- | --- | --- | --- |
|  | **Univariable** | | |  | **Multivariable** | | | |  | | **Univariable** | | |
| **Characteristic** | **OR** | **95% CI** | **p-value** |  | **aOR** | **95% CI** | **p-value** | |  | | **OR** | **95% CI** | **p-value** |
| Age (per year increase) | 1.32 | 1.25, 1.41 | <0.001 |  | 1.32 | 1.25, 1.41 | <0.001 | |  | | 1.41 | 1.32, 1.53 | <0.001 |
| Region of birth (UK/other vs Africa/Caribbean) | 2.44 | 1.27, 4.60 | 0.006 |  | 2.52 | 1.16, 5.42 | 0.018 | |  | | 1.29 | 0.72, 2.38 | 0.40 |
| CD4+ cell count (per two-fold increase) | 0.89 | 0.70, 1.15 | 0.35 |  |  |  |  | |  | | 0.93 | 0.70, 1.21 | 0.59 |
| HIV RNA ≥200 copies/mL | 1.68 | 0.81, 3.32 | 0.15 |  |  |  |  | |  | | 1.60 | 0.81, 3.35 | 0.19 |
|  |  |  |  |  |  |  |  | |  | |  |  |  |
| BMI ≥30 kg/m^2^ | 1.74 | 1.07, 2.92 | 0.030 |  | 1.70 | 0.92, 3.21 | 0.097 | |  | | 1.02 | 0.65, 1.62 | 0.92 |
| SBP >140 mm Hg | 5.14 | 3.17, 8.41 | <0.001 |  |  | - |  | |  | | 3.25 | 2.03, 5.31 | <0.001 |
| DBP >90 mm Hg | 2.35 | 1.44, 3.80 | <0.001 |  |  | - |  | |  | | 1.87 | 1.16, 3.07 | 0.011 |
| TC/HDL cholesterol >3.5 | 2.77 | 1.72, 4.47 | <0.001 |  |  | - |  | |  | | 1.00 | 0.64, 1.55 | 0.98 |
| Triglycerides >1 mmol/L | 4.03 | 2.37, 7.16 | <0.001 |  |  | - |  | |  | | 1.49 | 0.93, 2.40 | 0.10 |
| Smoking status (current/ex vs. never) | 3.31 | 1.65, 6.58 | <0.001 |  |  | - |  | |  | | 1.39 | 0.86, 2.28 | 0.19 |
|  |  |  |  |  |  |  |  | |  | |  |  |  |
| Diabetes mellitus | 16.7 | 9.11, 31.9 | <0.001 |  |  | - |  | |  | | 20.7 | 7.48, 86.1 | <0.001 |
| eGFR <60 mL/min/1.73m^2^ | 3.38 | 1.65, 6.87 | <0.001 |  |  | - |  | |  | | 40.5 | 8.71, 721 | <0.001 |
